# Characterisation of Patients With Chronic Obstructive Pulmonary Disease (COPD) From an Urban Municipality in the Northern Region of Portugal: A Cross-Sectional Study

**DOI:** 10.7759/cureus.59262

**Published:** 2024-04-29

**Authors:** Liliana Silva, Ângela Mota, Lara Lemos, Mariana Santos, Hélder Cunha, Tiago Maricoto, Patrício Costa, Miguel Padilha

**Affiliations:** 1 Rehabilitation Nursing, Matosinhos Local Health Unit, Matosinhos, PRT; 2 Technologies for Education and Simulation in Healthcare (Tech4edusim), Centro de Investigação em Tecnologias e Serviços de Saúde (CINTESIS), Porto, PRT; 3 Beira Ria Family Heath Unit, Unidade Local de Saúde da Região de Aveiro, Ílhavo, PRT; 4 Health Sciences Research Centre & UBIAir - Clinical & Experimental Lung Centre (CICS-UBI), University of Beira Interior, Covilhã, PRT; 5 Life and Health Sciences Research Institute (ICVS) School of Medicine, University of Minho, Braga, PRT; 6 Life and Health Sciences Research Institute (ICVS) ICVS'3, PT Government Associate Laboratory, Braga, PRT; 7 Faculty of Psychology and Educational Sciences, University of Porto, Porto, PRT; 8 Rehabilitation Nursing, Porto Higher School of Nursing, Porto, PRT; 9 Porto Higher School of Nursing, Centro de Investigação em Tecnologias e Serviços de Saúde - Rede de Investigação em Saúde (CINTESIS@RISE), Porto, PRT

**Keywords:** chronic obstructive pulmonary disease (copd), primary care, misdiagnoses, rehabilitation nurses, pulmonary rehabilitation

## Abstract

Background

Pulmonary rehabilitation (PR) is formally indicated to all COPD patients in groups B and E. It positively impacts dyspnoea, health-related quality of life and exercise tolerance, reducing admissions among people with chronic obstructive pulmonary disease (COPD) who have had a recent exacerbation and symptoms of anxiety and depression. There is limited access to PR programmes in Portugal, partially due to insufficient resources or referrals. This study aims to characterise COPD patients and assess whether they have criteria for PR programmes. Data from this study may provide strategic information for healthcare organisations to differentiate and innovate their response to COPD patients.

Methodology

A cross-sectional study was conducted in an urban municipality in the northern region of Portugal. The sample was randomly extracted from the national primary electronic health records. The sample size (n = 339) was determined considering the population of COPD patients in this region (N = 2818), a 95% confidence level and a margin of error of 5%.

Results

In this population, the prevalence of COPD is 1.8%. Furthermore, in this sample, 40% (n = 136) of people diagnosed with COPD have a formal indication to participate in PR programmes, although only 14.2% (n = 48) of these patients had access to PR.

Conclusion

COPD is probably underdiagnosed in this Portuguese region. Most COPD patients have eligibility criteria to be offered PR programmes, although most of them do not benefit from this vital treatment. Investing in community and home-based programmes may increase PR access, reducing acute exacerbation of chronic obstructive pulmonary disease (AECOPD) admissions.

## Introduction

Chronic obstructive pulmonary disease (COPD) is a leading cause of morbidity and mortality worldwide [1]. Persistent respiratory symptoms and airflow limitation occur due to airway and/or alveolar abnormalities [1].

COPD prevalence varies across different countries and often relates to tobacco smoking prevalence, and it is estimated that there is an underdiagnosis issue. For example, in Portugal, based on a national study of the Lisbon population in 2013, the estimated prevalence of people over 40 years old suffering from COPD is 14.2% [2]. This study's high prevalence figures, significantly exceeding prior research, have sparked controversy [3]. Nevertheless, in primary care electronic records, only 1.6% of people are diagnosed with COPD [4]. The Global Initiative for Chronic Obstructive Lung Disease (GOLD) states that the worldwide prevalence is around 10.4%, so the underdiagnosis of COPD remains an issue [1].

The main concern with COPD underdiagnosis and misdiagnosis lies in the fact that some people with characteristic symptoms, such as dyspnoea, functional limitation and cough, with or without sputum, are not receiving adequate care. On the other hand, misdiagnosis can also represent a problem that leads to patients receiving incorrect treatment and an overuse of resources [1].

The most cost-effective non-pharmacological treatment for COPD is pulmonary rehabilitation (PR) [5]. PR is a comprehensive intervention that includes but is not limited to exercise training, education, self-management intervention aiming at behaviour change and promoting long-term adherence to health-enhancing behaviours [1,6,7].

PR is an essential treatment for all people with COPD in groups B and E (symptomatic and/or exacerbators) [1,6]. It positively impacts dyspnoea, health-related quality of life and exercise tolerance. In addition, it reduces the number of hospital admissions among people with COPD who have had a recent exacerbation and reduces symptoms of anxiety and depression [1,8].

There is limited access to PR programmes in Portugal, partially due to, but not limited to, insufficient resources or referrals [6,9-12]. In Portugal, around 2% of COPD patients have access to PR programmes [10].

In this municipality, there are centre-based programmes conducted in specialised centres and community-based programmes delivered in primary care.

It is essential to know the patients' characteristics to develop alternatives to centre-based PR programmes and increase the offer to such an essential treatment.

This study was conducted in an urban area with a PR centre based at this region's hospital, providing a structured PR programme. Since 2021, due to the necessity of alternatives to centre-based PR, a community and home-based programme delivered by four primary care practices, was integrated into the existing one, creating a structured and unified PR programme. After referral by a clinician, a multidisciplinary team discusses the patient's case deciding which approach (centre-based or community/home-based) suits the patient better, taking into account clinical or social factors, enhancing adherence to treatment.

This study aims to characterise COPD patients from an urban area in northern Portugal, using sociodemographic, clinical variables and disease classification to assess whether they have criteria for PR programmes according to the guidelines and the proportion of patients with access to PR in this region. Data from this study may provide strategic information for healthcare organisations to differentiate and innovate their response to COPD patients.

## Materials and methods

After receiving authorization and ethical approval from the Ethics Committee of the Matosinhos Local Health Unit, Portugal (81/CES/JAS - 09/06/2021), this research was conducted in an urban municipality in the northern region of Portugal.

Design and setting

We conducted a cross-sectional study in an urban area. In this region, the population diagnosed with COPD corresponds to 2.818 patients [4]. A sample of COPD patients was randomly extracted from the national primary care electronic health records (EHRs). All general practitioners' diagnoses are registered and coded as R95, according to the International Classification of Primary Care, 2nd edition (ICPC-2).

The sample size (n = 339) was determined considering the population under study (2,818), a 95% confidence level, a worst-case scenario for the proportion variance (p = 0.5) and a margin of error of 5%. The sample size was calculated using the OpenEpi software - Toolkit Shell for Developing New Applications [13].

Data collection procedures

Data were accessed through EHRs following ethical research standards and ensuring anonymity. We randomly selected a sample of 339 individual files from a list of all COPD patients to be evaluated using a random sequence generator on Microsoft Excel (Microsoft Corporation, USA).

According to the GOLD 2024 ABE scheme [1], we searched for COPD clinical classification in the EHRs registered by patients' general practitioners. Participants' records were screened to assess the presence of eligibility criteria for PR programmes and whether previous PR programmes were already implemented. Patients were considered eligible for PR if they had COPD classification B or E (previously C and D, according to the GOLD 2022)[14] and absence of clinical contraindications, such as comorbidities that limit exercise training: ischaemic cardiopathy, unstable angina, severe aortic stenosis, hypertrophic cardiomyopathy, uncontrolled arrhythmia, decompensated congestive heart failure, uncontrolled diabetes, severe cognitive dysfunction or severe psychiatric disease interfering memory and adherence [15].

Lung function was also evaluated, particularly the percentage of forced expiratory volume in the 1st second (FEV_1_%) and the FEV_1_/FVC ratio (recorded as <70% or >70%) after a bronchodilation test (BD), retrieved from the patients' spirometry records [1,16]. Additional information included the number of emergency department visits and admissions due to acute exacerbations of COPD in the last 12 months (admissions for other causes were not considered) and if they are under follow-up by a pulmonologist. We categorised the number of admissions into "No admissions of acute exacerbations of COPD (AECOPD)" and "At least one admission of AECOPD," following the rationale for the GOLD criteria at patient characterisation [1].

Statistical analysis

The statistical tests were performed using IBM SPSS Statistics for Windows, version 28.0 (released 2021, IBM Corp., Armonk, NY), and results were considered significant for p < 0.05. Categorical variables were described through absolute frequencies (n) and relative frequencies (%). Continuous variables were described using mean and standard deviation.

We investigated the association between previous participation in the PR programme and hospital admission for acute exacerbation of chronic obstructive pulmonary disease (AECOPD) using Fisher's exact test. To compare continuous variables in different groups, we used the independent samples Student's t-test and, for dichotomous variables, the Fisher's exact test.

We also did a logistic regression to identify factors related to access to PR and calculated the odds ratio of having an AECOPD, comparing those who had access to PR and those who did not.

## Results

The collected sample is representative of this population regarding gender (33% females) and age categories (18-64, 35%; 65 to 75, 34%; +75, 31%). These proportions are similar to the Portuguese population (37% females; 18-64, 33%; 65-75, 30%; +75, 37%) [13].

Table 1 shows the characteristics of the total patient sample and the sample eligible for PR. The individual's average age is 69.5 years (SD ± 1.6) (66.4%, n = 225, of the total sample with more than 65 years old). The mean FEV_1_% is 75.5% (SD ± 20.3). In addition, 29% (n = 100) of these individuals regularly visit a pulmonologist in secondary healthcare.

**Table 1:** Characteristics of the total sample of patients and the sample eligible for pulmonary rehabilitation (PR). The data has been represented as N, %. a: differences in n due to missing values; b: without mention in the electronic records; c: AECOPD (cute exacerbation of chronic obstructive pulmonary disease (COPD)

	Total (n = 339)	Eligible to PR (n = 136)
Age (years), n (%)		
18-64	114 (33.6)	42 (30.9)
65-74	99 (29.2)	37 (27.2)
≥75	126 (37.2)	57 (41.9)
Females, n (%)	113 (33.3)	46 (33.8)
FEV_1_ % predicted, n (%)^a^		
≥80	113 (33.3)	41 (30.1)
50-79	145 (42.8)	66 (48.5)
30-49	25 (7.4)	8 (5.9
<30	1 (0.3)	--
GOLD classification, n (%)^a^		
A	135 (39.8)	--
B	118 (34.8)	104 (76.5)
E	38 (11.2)	32 (23.6)
Unclassified^b^	48 (14.2)	--
Absolute exclusion criteria presence, n(%)	31 (9.1)	--
Admissions in the last year^c^, n (%)		
0	314 (92.6)	118 (86.8)
≥1	25 (7.4)	18 (13.2)
Emergency department visits in the last year^c^, n(%)		
0	291 (85.8)	104 (76.5)
1	31 (9.1)	19 (14)
2	12 (3.5)	11 (8.1)
≥3	5 (1.5)	2 (1.5)
Previous PR attendance (centre-based or community-based), n (%)	32 (9.4)	21 (15.4)

Acute exacerbations of COPD

Concerning the number of AECOPD, the eligible population for PR had more emergency visits and hospital admissions. Among people with at least one admission for AECOPD, 76% (n = 19) never had access to PR. A significant association between access to PR and admissions for AECOPD was found (p = 0.021, Fisher exact's test) (Table 2). No statistically significant associations were found between FEV1% and the time since the last PR programme (r(24) = 0.085, p = 0.692). 

**Table 2:** Access to PR and acute exacerbation of chronic obstructive pulmonary disease (AECOPD) admissions The data are represented as N, %. p < 0.05.

	No admissions AECOPD (n=314)	≥1 Admission AECOPD (n=25)
No access to PR n(%)	288(91.7)	19(76.0)
Access to PR n(%)	26(8.3)	6(24.0)

Spirometric values

Eighteen per cent (n = 62) of the patients had an FEV_1_/FVC ratio above 70% and therefore, without clear diagnostic criteria for airway obstruction. Spirometric measures were unavailable in 18% of the patient's records. Thirty-five per cent (n = 117) had an FEV_1_% record in the last three years.

Pulmonary rehabilitation

In this sample, 40% (n = 136) of individuals formally meet the criteria to qualify for PR programmes. However, in 14.2% (n = 48) of COPD diagnoses, categorisations have not been described by their general practitioners, and the eligibility criteria could not be assessed due to a lack of data (CAT or mMRC score). Data comparing eligible and not eligible PR groups show a slight difference concerning age, in which 69.1% (n = 94) of the eligible individuals are over 65 (Table 1). PR-eligible patients generally had more exacerbations (emergency visits and hospital admissions), but 76.5% (n = 104) are characterised as GOLD B, and 78.6% (n = 107) have mild to moderate obstructions according to the FEV_1_ value.

The history of previous participation in a PR programme among the total population is 9.4% (n = 32), either centre-based or community-based. Among eligible patients for PR, 15% (n = 21) had received it, and 67% (n = 16) had repeated the programme at least once.

Logistic regression did not have statistical power to find the factors related to PR access or referral. Moreover, the calculated odds ratio of having an AECOPD, comparing those who had access to PR and those who had not, did not show statistical significance.

## Discussion

Main findings

In our study, COPD prevalence based on the EHRs was lower than expected when compared to the estimated national prevalence of 14% in people over 40 [2,4]. Low prevalence can be related to underdiagnosis and/or under-recording/underreporting. According to data extracted from primary health care national EHRs, the prevalence of COPD diagnosis in this northern Portuguese area is 1.8%, slightly higher than the one referring to the Portuguese population, where the value is 1.6% [4].

According to the GOLD standards [1], eligible patients for PR programmes have mild to moderate COPD. Community- or home-based programmes may be essential to increase PR access, specifically in this group of patients. On the other hand, hospital-based programs may reach individuals with more severe COPD stages and with clinical contraindications to perform this intervention in the community.

One of the findings of this study points out an insufficient accuracy of patient records, lacking the characterisation of COPD according to the severity of symptoms and functional limitations. This means that disease classification remains, in part, documented according to lung obstruction instead of limitations caused to the patient.

Portuguese EHR lacks a specific area for recording data from follow-up programmes for patients with chronic respiratory diseases, although it exists for other diseases, such as diabetes and hypertension. The nonexistence of this specific area in the EHR probably contributes to less clinical data quality from patients with CRD and less awareness of health professionals to patients with asthma or COPD.

Community- and home-based programmes are as effective as the ones delivered in specialised centres, mainly increasing functional capacity and quality of life, even with scarce equipment [17].

According to previous reports from Portugal and around the world, our region stands out for having significantly higher access to PR programmes [6,9,10]. However, looking at patients with eligibility criteria only, the access rate is 15.4%, which is still considerably low. Nevertheless, this region also has community and home-based programmes, which may promote easier access in the coming years.

Another finding of this study was the existence of COPD misdiagnoses in primary care, where 18% of the patients were labelled with COPD having FEV_1_/FEV >70% and 18% of COPD diagnoses were documented without a spirometry record. It is possible that these last 18% without Tiffenou value, could have a confirmatory spirometry performed elsewhere (outside the National Health System) but not available and registered in the electronic records.

The above findings influences patients’ clinical outcomes and healthcare services and data validity for research.

Strengths and limitations

This study, whose sample reflects good practices, characterises a COPD population from an urban area in northern Portugal, with a simple random sampling process, based on a random number generator. It represents the general population considering that gender and age group distribution is similar to the Portuguese population.

A significant limitation of this study is that there is not a clear, consistent record of CAT or mMRC in the Portuguese electronic records for all COPD diagnoses. This information had to be extracted from the general register, where clinicians would document it occasionally, although it was limited to the record-keeping detail of each general practitioner.

According to electronic records, our data suggest that only 64% of COPD diagnoses in primary health care are accurate within international guidelines and with objective measures of airway obstruction [1,16]. Nevertheless, 18% of patients without spirometric records may have performed it elsewhere. Consequently, we do not have clear evidence to confirm or exclude the diagnosis.

Only the COPD diagnoses encoded with the ICPC R95 were considered in this study. Therefore, our results may not represent all the estimated COPD population, considering the potential for underdiagnosis and imprecision of the currently diagnosed patients.

Data were collected during the COVID-19 pandemic, which may have influenced COPD patients' behaviour concerning the number of emergency services and admissions.

We only considered an episode of AECOPD in the presence of an emergency department visit and/or an admission registered due to AECOPD, so we may not have taken into account some AECOPD events treated in primary care settings.

Implications for clinical practice and stakeholders

Considering the Portuguese primary care system overview, making community-based PR programmes available to patients becomes a key topic in COPD management and trained health professionals in rehabilitation (such as rehabilitation nurses) can play a crucial role in promoting access, taking into account its potential benefits [18,19].

According to this region's management data, the average cost of a hospital admission for COPD exacerbation (per day) is €229.77, and the number of admission days is, on average, 9.3 [20]. In addition, the average emergency service cost for a COPD exacerbation is €153.90 [20]. According to the characteristics of the population in the study, increasing access to PR through community and home-based programmes can reduce emergency department visits and admissions for AECOPD, as well as costs inherent to these.

Likewise, a precise diagnosis and patient categorisation are of paramount importance, since offering misdiagnosed patients a medical speciality referral for pharmacological or non-pharmacological treatments may involve the overuse of different resources, affecting the overall capacity of the healthcare system.

## Conclusions

Forty per cent of patients met the criteria for PR programmes based on the GOLD classification (stage B or E) and the absence of clinical contraindications. However, for 14% of patients, COPD classification data were missing, making PR eligibility unclear.

The access to this essential treatment in this municipality is 15.4%, which is still considerably low but higher than the available data from the Portuguese, and two-thirds repeated the program at least once.

Regarding the known barriers to PR and the percentage of people with COPD who do not uptake or complete this essential treatment, investing in community and home-based programmes may increase PR access, reducing AECOPD admissions.
